# Evaluation of the Age- and Sex-Related Changes of the Osteogenic Differentiation Potentials of Healthy Bone Marrow-Derived Mesenchymal Stem Cells

**DOI:** 10.3390/medicina57060520

**Published:** 2021-05-22

**Authors:** Hyun-Jin Lee, Hyuna Lee, Chae-Bin Na, In-Seok Song, Jae-Jun Ryu, Jun-Beom Park

**Affiliations:** 1 Department of Periodontics, College of Medicine, The Catholic University of Korea, Seoul 06591, Korea; hyunjinlee0423@gmail.com (H.-J.L.); hya602@naver.com (H.L.); cebina4959@naver.com (C.-B.N.); 2 Department of Oral and Maxillofacial Surgery, Korea University Anam Hospital, Seoul 02841, Korea; 3 Department of Prosthodontics, Korea University Anam Hospital, Seoul 02841, Korea; koprosth@gmail.com

**Keywords:** age factors, bone marrow, cell differentiation, sex, stem cells

## Abstract

*Background andObjectives:* Human bone marrow-derived mesenchymal stem cells (BMSCs) are promising sources for cell-based regenerative therapy. The purpose of the present study was to elucidate the roles of age and sex on the cellular viability and osteogenic potential of BMSCs cultured in osteogenic media. *Materials and Methods:* Human BMSCs were isolated and expanded from 3 age groups—20s, 30s, and 50s—from both sexes. The total number of aspirates was ten, and each subgroup had five for 20s (two females and three males), three for 30s (one female and two male), and two for 50s (one female and one male). Analyses of the cell morphology, the cell viability, the expression of the stem cell marker SSEA-4, the secretion of human vascular endothelial growth factor (VEGF), the expression of Runx2 and collagen I, the metabolic activity, and the formation of mineralization nodules were performed. *Results:* No significant differences were found in the cell viability of human BMSCs cultured in osteogenic media among the different age groups. There were no significant differences in the expression of SSEA among the age groups or between males and females. There were no significant differences in the secretion of human VEGF between males and females. No significant differences in Runx2 or collagen I expression were noted by age or gender. Moreover, no significant differences were shown in osteogenesis by alizarin red staining. *Conclusions:* The human BMSCs showed no age-related decreases in cellular viability or osteogenic differentiation potential.

## 1. Introduction

Human mesenchymal stromal/stem cells are considered invaluable resources of replacements for injured and elderly cells, and they are widely documented for their regenerative potentiality for tissue engineering and cell therapy [1,2]. Being the most exploited source of mesenchymal stem cells, bone marrow-derived mesenchymal stem cells (BMSCs) are of great importance due to their ability to support other progenitors of the immune and blood system [3], engage in the repair system of extramedullary tissues [4], and modulate bone regulation through paracrine stimulus [5,6].Cellular aging is of great importance for experimental conditions and the therapeutic effectiveness of tissue engineering [7]. The cells experience a replicative aging process, which results in senescence by shortening the cells’ telomeres when cells are subcultured [8,9]. Therefore, early-passage cells have been used in experiments in vivo and in vitro. However, it is still unclear whether there are age- or sex-related differences in BMSCs’ osteogenic function, as well as in the replicative aging of MSCs. There is still controversy about the roles of age and gender in the osteogenic differentiation ability of human derived BMSCs. Many studies have demonstrated significant reductions due to aging [8,9,10]. In a previous report, aging led to a biased differentiation in adipogenesis at the cost of osteogenesis, leading to a decreased bone formation [10]. It was also shown that the regenerative capacity of BMSCs was significantly influenced by age, and young BMSCs produced higher functional outcomes [11]. However, a couple of reports showed no differentiation among young and old donors [1,12] Likewise, several studies have reported differences in osteogenesis between sexes [13,14]. A rodent study revealed that osteogenesis is sexually dimorphic, with proliferation and osteoblastic differentiation being superior in male mice than in female mice [15]. However, another study reported contradictory results and found that female mice were more susceptible to the rosiglitazone-derived effects on osteogenesis, as compared to males [16].

Therefore, it is valuable to elucidate age- or sex-associated differences in the osteogenic potential of BMSCs to estimate or standardize their therapeutic potential. The target of this study is to elucidate the roles of age and sex in cellular viability, the expression of the stem cell marker SSEA-4, the secretion of human vascular endothelial growth factor (VEGF), Runx2, and collagen mRNA, and the Runx2 and collagen I protein expression of human BMSCs cultured in osteogenic media.

## 2. Materials and Methods

### 2.1. Bone Marrow-Derived Mesenchymal Stem Cells

The Institutional Review Board of Seoul St Mary’s Hospital, College of Medicine, The Catholic University of Korea reviewed and approved the present work (KC18SESI0083, 20 February 2018), and all of the experimental schemes used were performed according to the relevant guidelines. Human BMSCs (Catholic MASTER Cells) were gained from the Catholic Institute of Cell Therapy (CIC, Seoul, South Korea). Human bone marrow aspirates were obtained from the iliac crest of healthy donors. Human BMSCs were isolated and expanded from 3 age groups—20s, 30s, and 50s—from both sexes. The total number of aspirates was ten, and each subgroup had five for 20s (two for female and three for male groups), three for 30s (one for female and two for male groups), and two for 50s (one for female and one for male groups). The isolation and propagation of the BMSCs were performed following a previously reported method [17]. The Catholic Institute of Cell Therapy has ensured that all the samples showed CD73 and that the CD 90 expression was >90% positive. Moreover, the Catholic Institute of Cell Therapy tested that CD31, CD 34, and CD 45 were >90% negative. The cells were plated on a culture dish, and the cells that were detached from the dish were eliminated. The culture medium was refreshed every 2 or 3 days, and the BMSCs were nurtured with 95% air and 5% CO_2_ at 37 °C in the incubator.

### 2.2. Cellular Morphology and Determination of Cell Viability

First, 1.0 × 10^6^ BMSCs with passage 2 were used, and the cells were plated at a seeding density of 2.0 × 10^3^/96 well plate and 2.0 × 10^4^/24 well plate. BMSCs were grown in an osteogenic medium, which is composed of an alpha-minimal essential medium (α-MEM, Gibco, Grand Island, NY, USA) comprising 200 mM of L-Glutamine (Sigma-Aldrich Co., St. Louis, MO, USA), 10 mM of ascorbic acid 2-phosphate (Sigma-Aldrich Co.), 100 μg/mL of streptomycin (Sigma-Aldrich Co.), 15% fetal bovine serum (Gibco), 100 U/mL of penicillin, 2 mg/mL of glycerophosphate disodium salt hydrate, and 38 μg/mL of dexamethasone. Inverted microscopy was used for the evaluation of the morphology of the tested stem cells (CKX41SF, Olympus Corporation, Tokyo, Japan) on Days 1 and 3. The differences among the 20s, 30s, and 50s groups were analyzed. The experimental repeats were performed in triplicate.

The BMSCs was qualitatively analyzed with a LIVE/DEAD Kit assay (Molecular Probes, Eugene, OR, USA) for the viability. Cultured BMSCs were washed twice using a culture medium and then suspended in 1 mL of α-MEM containing 2 μL of a 50 mM calcein acetoxymethyl ester working solution and 4 μL of 2 mM ethidium homodimer-1 for 30 min at room temperature. The BMSCs were stained with ethidium homodimer-1 and calcein acetoxymethyl ester, then examined under a fluorescence microscope (Axiovert 200; Zeiss, Germany) on Day 4.

A viability test was performed on Days 1 and 3 for the quantitative analysis. The WST-8 (2-(2-methoxy-4-nitrophenyl)-3-(4-nitrophenyl)-5-(2,4-disulfophenyl)-2H tetrazolium, monosodium salt) (Cell Counting Kit-8; Dojindo, Tokyo, Japan) was supplemented to the culture medium, and the BMSCs were incubated for 1 h at 37 °C. The absorbance of the BMSCS was calculated at 450 nm with a microplate reader using a spectrophotometer (BioTek, Winooski, VT, USA).

### 2.3. Immunofluorescence

An immunofluorescent assay was performed using human SSEA-4 antibody (mab1435, R&D Systems, Inc., Minneapolis, MN, USA) on Day 3. The BMSCs were fixated, permeabilized, blocked, then reared in the incubator with SSEA-4 primary antibody. The cultures were incubated with secondary antibody conjugated with fluorescein isothiocyanate (F2761, Abcam, Cambridge, UK), followed by staining with 4’,6-diamidino-2-phenylindole. A fluorescence microscope (Axiovert 200) was used for the analysis.

### 2.4. Secretion of Human Vascular Endothelial Growth Factor from the BMSCs

The secretion of human VEGF was determined on Day 2 and Day 3 using a commercially available kit (Quantikine^®^ ELISA, cat# DVE00, R&D Systems, Inc., Minneapolis, MN, USA). All reagents and samples were prepared according to the manufacturer’s recommendations.

### 2.5. Total RNA Extraction and Quantification Using a Real-Time Polymerase Chain Reaction

The isolation and purification of Total RNA was performed using a GeneJET RNA Purification Kit (Thermo Fisher Scientific, Inc., Waltham, MA, USA) on Day 2. A template containing 1 ng of total RNA was utilized for reverse transcription using SuperiorScript II Reverse Transcriptase (Invitrogen, Carlsbad, CA, USA), and the determination of quantity was conducted using a spectrophotometer (ND-2000, Thermo Fisher Scientific, Inc.) with an absorbance ratio at 260/280 nm on Day 2. A quantitative real-time polymerase chain reaction (qPCR) was conducted to analyze the mRNA expression by SYBR Green Real-Time PCR Master Mixes (Enzynomics, Daejeon, Republic of Korea). The primers used for the qPCR were designed as follows: Collagen I Forward 5′–TCA TGG CCC TCC AGC CCC CAT3′; and Reverse 5′–ATG CCT CTT GTC CTT GGG GTT C–3′; Runx-2 Forward 5′–AAT GAT GGT GTT GAC GCT GA–3′; and Reverse 5′–TTG ATA CGT GTG GGA TGT GG–3′. The mRNA levels were expressed as fold changes, which were normalized to β-actin.

### 2.6. Western Blotting Analysis

The BMSCs were rinsed twice using iced phosphate-buffered saline (PBS, Welgene, Daegu, Korea) and solubilized in an RIPA lysis buffer (Thermo Fisher Scientific, Inc.) with protease inhibitors (PPI1015, Quartett, Bern, Germany) on Day 2 for 30 min. The centrifugation of lysate was undertaken at 4 °C for 10 min at 13,000 rpm. Next, the sample underwent electrophoresis with sodium dodecyl sulfate polyacrylamide gel (Mini-PROTEAN^®^ TGX™ Precast Gels, Bio-Rad), then transferred to polyvinylidene difluoride membranes (IB24002, Immun-Blot^®^, Bio-Rad, Hercules, CA, USA) using a transfer apparatus (iBlot^®^ 2 Transfer Stacks, Bio-Rad). The membrane was immunoblotted by corresponding antibodies and enhanced chemiluminescent detection kits. The quantification of the expression levels of the proteins, including Runx2, collagen I, and GAPDH, was performed with image analysis and processing software (ImageJ, National Institutes of Health, Bethesda, MD, USA).

### 2.7. Evaluation of the Adenosine 5-Triphosphate Assay

The adenosine 5-triphosphate assay (Sigma-Aldrich Co.) was measured using a commercially available kit, following the manufacturer’s instruction. In short, an adenosine 5-triphosphate assay mix solution was added to a reaction vial. Then, the sample was added to the assay vial, and measurements were performed.

### 2.8. Alkaline Phosphatase Activity

The level of the alkaline phosphatase activity was used to assess the osteogenic differentiation [18]. Commercially available kits (K412-500, BioVision, Inc., Milpitas, CA, USA) were used to evaluate the level of alkaline phosphatase activity at the absorbance at 405 nm.

### 2.9. Alizarin Red S Staining

BMSCs groups consisting of 2 × 10^4^ cells were sowed on 24-well plates and grown with an osteogenic medium containing 2 mg/mL of glycerophosphate disodium salt hydrate, 38 μg/mL of dexamethasone, 10 mM of ascorbic acid 2-phosphate (Sigma-Aldrich Co.), 200 mM of L-glutamine (Sigma-Aldrich Co.), and 15% fetal bovine serum (Gibco). On Days 8 and 16, the cells were rinsed twice with PBS (Welgene), followed by fixation with 4% paraformaldehyde, then cleansed twice with deionized water. The sample was stained using alizarin red S (Sigma-Aldrich Co.) at room temperature for 30 min. 

To eliminate the staining of non-specific binding, the cells were rinsed three times with deionized water. The solubilization of bound dye was performed using 10 mM of sodium phosphate comprising 10% cetylpyridinium chloride, then quantitated at 562 nm by a spectrophotometer. The inverted microscope was utilized for morphological evaluation (CKX41SF, Olympus Corporation, Tokyo, Japan). A quantitative analysis of alizarin red S was accomplished using image analysis and processing software (ImageJ, National Institutes of Health, Bethesda, MD, USA).

### 2.10. Statistical Analysis

The statistics are denoted as the means ± standard deviations. The effect of age and sex on the dependent variables was analyzed using regression analysis. A test of normality by a Shapiro-Wilk test and a one-way analysis of variance with a post hoc Tukey test was conducted to examine the differences between the age groups using a software program (SPSS 12 for Windows, SPSS Inc., Chicago, IL, USA). Kruskal Wallis tests were used for the comparison between the age groups, when the results were not normally distributed. The differences between males and females were analyzed by a *t*-test. Mann-Whiteny U tests were used for the comparison between the males and females when the data were not normally distributed. The data were considered statistically significant at a *p*-value below 0.05.

## 3. Results

### 3.1. Cellular Morphology and Cell Viability

**Figure 1:**
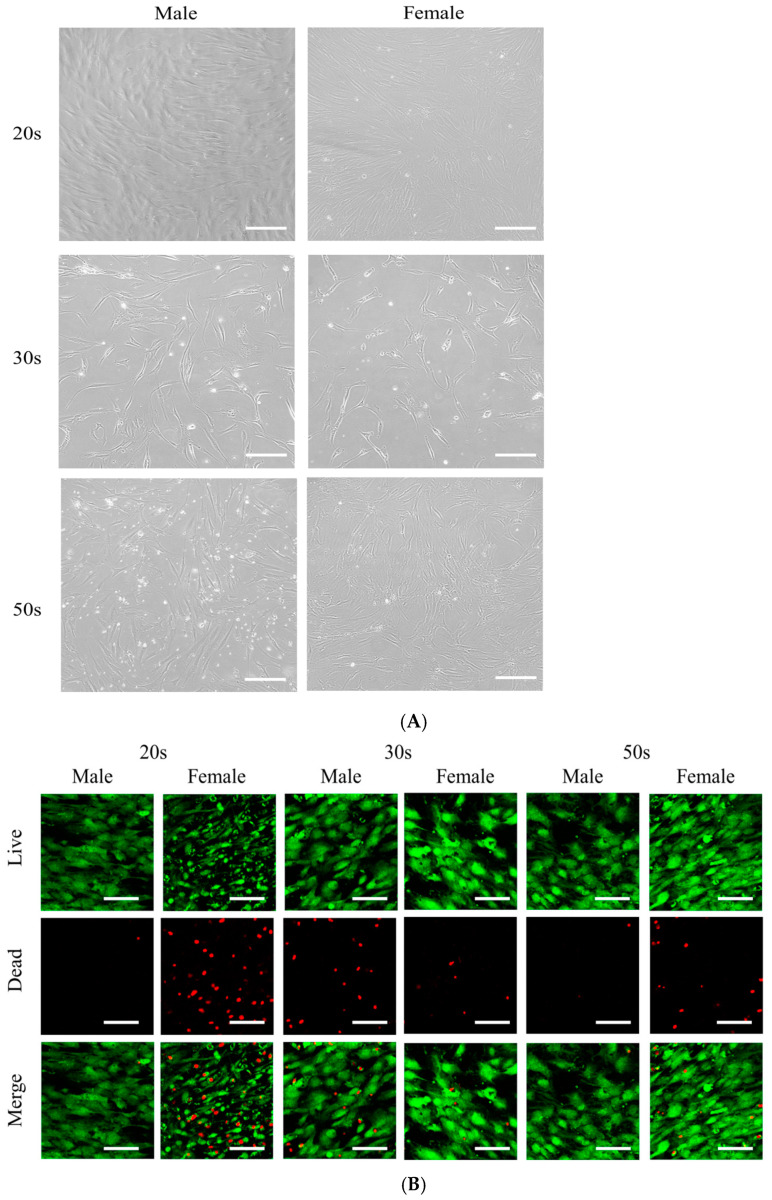

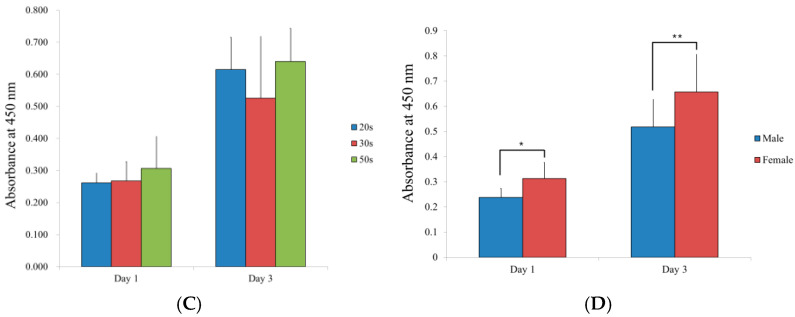
(**A**) Evaluation of the BMSCs’ morphology on Day 1 in osteogenic media (original magnification 100×). The scale bar indicates 200 μm. (**B**) Qualitative cellular viability results under a confocal microscope on Day 4. Live images, dead images, merged images, and central images are provided. The scale bar indicates 100 μm. (**C**) Cellular viability using CCK-8 assay on Days 1 and 3 among the age groups. (**D**) Cellular viability using a CCK-8 assay on Days 1 and 3 in males and females. * Statistically significant differences were noted when compared with the males’ results on Day 1 (*p <* 0.05). ** Statistically significant differences were noted when compared with the males’ results on Day 3 (*p <* 0.05).

The BMSC groups showed shapes resembling fibroblasts on Day 1 (Figure 1A). The number of cells were smaller in the 50s female and 50s males when compared with the 20s groups. It seems that there are lower number of cells in the 30s groups, especially in the 30s female group. The cellular viability evaluated using a LIVE/DEAD Kit assay was shown in Figure 1B. Most of the stem cells showed a green fluorescent signal, indicating a live status. However, there are more dead cells in the 20s female, 30s male, and 50s female groups. The results of CCK-8 on Days 1 and 3 are revealed in Figure 1C. The results of cellular viability showed no significant differences among the different age groups (Figure 1C). However, there were significant differences in terms of the cellular viability between the male and female groups on Days 1 and 3 (*p <* 0.05) (Figure 1D).

### 3.2. Immunofluorescence and Secretion of Human Vascular Endothelial Growth Factor from the BMSCs

**Figure 2:**
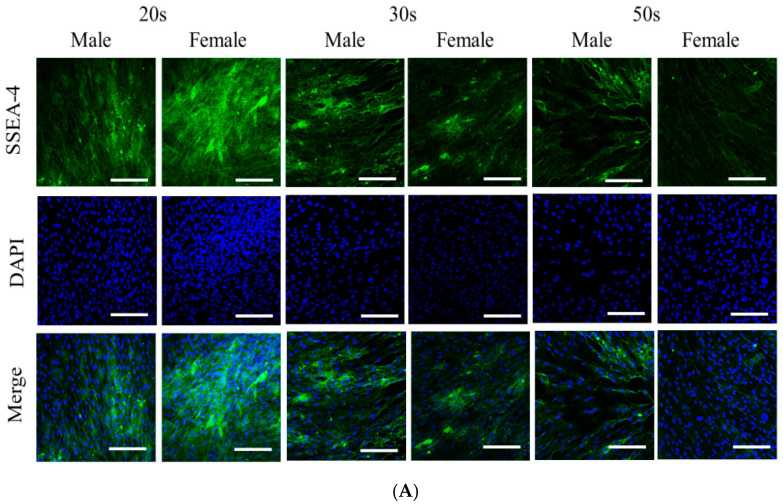

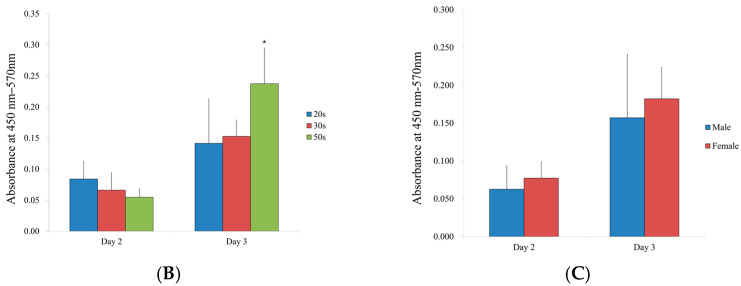
(**A**) Expression of the stem cell marker SSEA-4. (**B**) Secretion of vascular endothelial growth factors from BMSCs by different age groups. * Statistically significant differences were noted when compared with the results of the 20s group on Day 3. (**C**) Secretion of vascular endothelial growth factors in males and females.

Figure 2A shows the results of staining the BMSCs with SSEA-4, as shown in Figure 2A. All of the age groups were well stained with the SSEA-4 markers, and any significant differences were not found between the male and female groups. Secretions of vascular endothelial growth factors from the BMSCs were noted in all of the groups on Days 2 and 3 (Figure 2B,C). No significant differences in VEGF secretion were noted on Day 2 among the age groups, but significant differences were noted between the 20s and 50s groups on Day 3 (*p <* 0.05). There were no significant differences between males and females on Days 2 and 3.

### 3.3. Validation of mRNA Expression by qPCR

**Figure 3:**
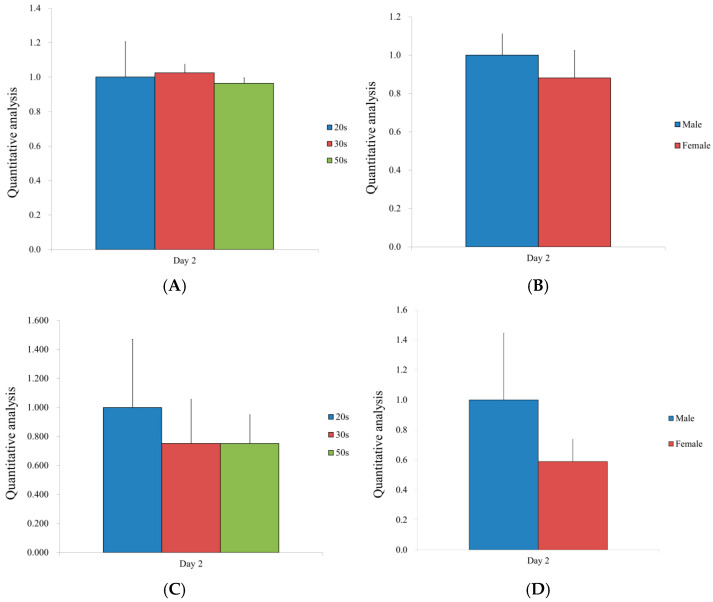
(**A**) Runx2 gene expression of hBMSCs according to age. (**B**) Runx2 gene expression of hBMSCs according to sex. (**C**) Collagen I gene expression of hBMSCs by age. (**D**) Collagen I gene expression of hBMSCs by sex.

The detection of Runx2 and collagen type I mRNA expressions was performed by qPCR. The levels of mRNA were normalized to glyceraldehyde 3-phosphate dehydrogenase (GAPDH) and stated as a fold change (Figure 3). There were no significant differences in the expression levels of Runx2 among the different age groups or between males and females (Figure 3A,B). Similarly, no statistical differences were noted between males and females, or among the different age groups (Figure 3C,D).

### 3.4. Western Blot

**Figure 4:**
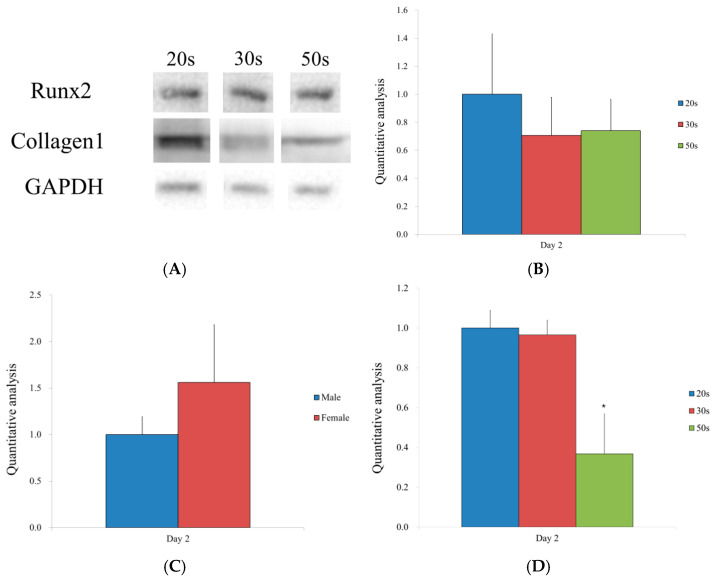
(**A**) Western blot analysis to detect the protein expressions of Runx2, collagen I, and GAPDH by hBMSCs. The experimental lanes were run in tandem. (**B**) Quantitative analysis of Runx2 levels by age. (**C**) Quantitative analysis of the Runx2 expression by sex. (**D**) Quantitative analysis of the collagen I expression by age. * Significant differences were noted when compared with the results of the 20s group.

The protein expression levels of Runx2 and collagen I are shown in Figure 4A. The relative expression of Runx2 for the 20s, 30s, and 50s groups was 1.000 ± 0.432, 0.707 ± 0.271, and 0.740 ± 0.223, respectively (Figure 4B). There were no significant differences in Runx2 levels among the different age groups. Likewise, there was no significant difference between the sexes on Day 2 (Figure 4C). The relative expression of collagen I for the 20s, 30s, and 50s groups was 1.000 ± 0.088, 0.965 ± 0.074, and 0.368 ± 0.084, respectively (Figure 4D). In particular, there was a reduction in protein expression in the 50s group, compared to the 20s and 30s groups (*p <* 0.05).

### 3.5. Evaluation of the Adenosine 5-Triphosphate Assay

**Figure 5:**
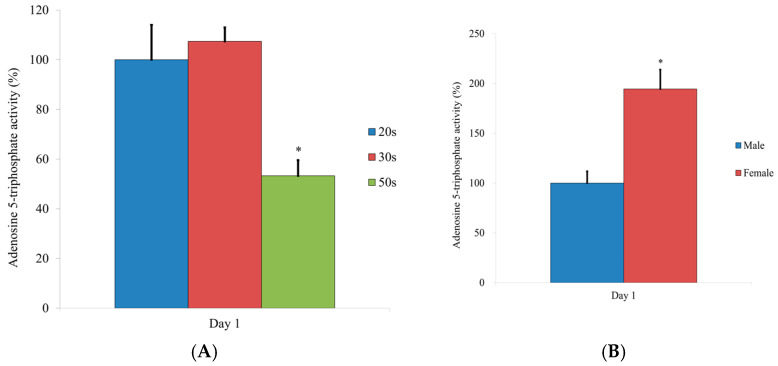
Results of the adenosine 5-triphosphate assay at Day 1. (**A**) Results from the different age groups. * Statistically significant differences were noted when compared with the results of the 20s group. (**B**) Results on Day 1 by sex. * Significant differences were noted when compared with the male group.

Figure 5 shows the results for the adenosine 5-triphosphate assay. Statistically significant differences were noted when compared with the results of the 20s group on Day 1 (*p <* 0.05) (Figure 5A). There were significant differences between the male and female groups (*p <* 0.05) (Figure 5B). 

### 3.6. Alkaline Phosphatase Activity

**Figure 6:**
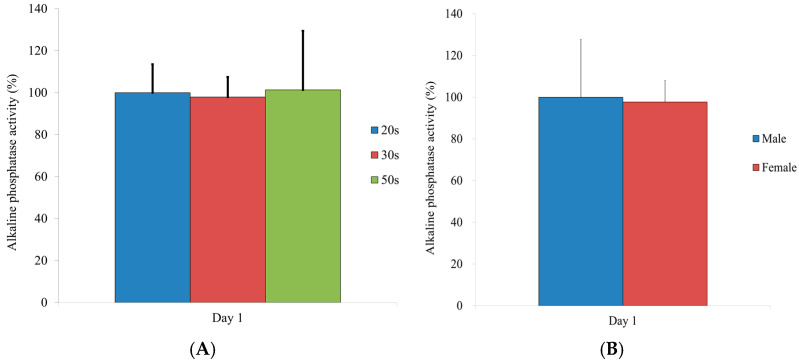
Alkaline phosphatase activity on Day 1. (**A**) Results from the different age groups. (**B**) Results on Day 1 by sex.

Figure 6 shows the results for the alkaline phosphatase activity. No significant differences were noted between the different age groups (*p* *>* 0.05). There were no statistical differences between males and females (*p* *>* 0.05).

### 3.7. Osteogenic Differentiation Potential by Alizarin Red S Staining

**Figure 7:**
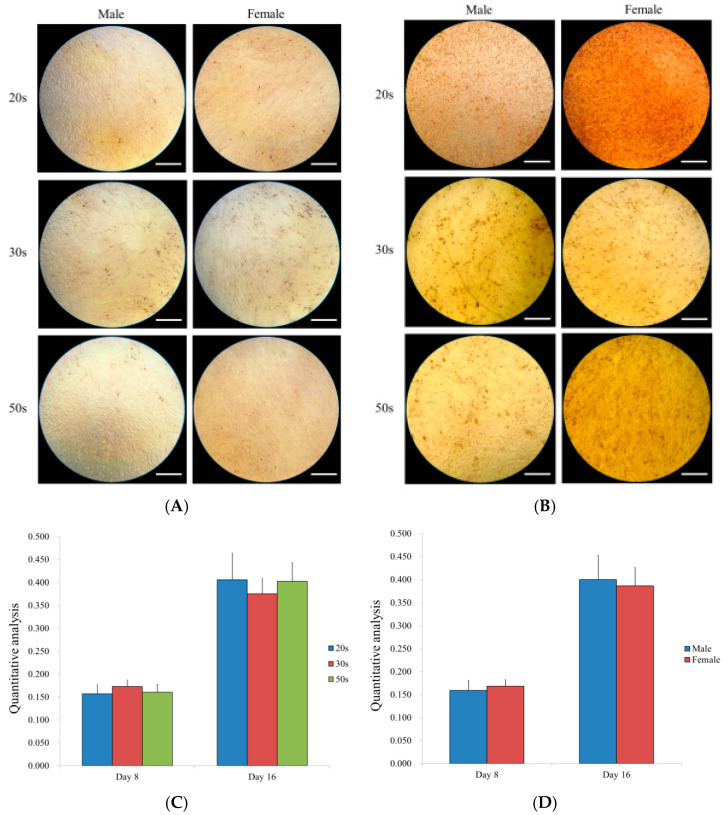
(**A**) Results of alizarin red S staining on Day 8 (original magnification 100×). The scale bar indicates 400 μm. (**B**) Results of alizarin red S staining on Day 16. The scale bar indicates 400 μm. (**C**) Quantitative results of the mineralization assay on Days 8 and 16 among the different age groups. (**D**) Quantitative results of the mineralization assay on Days 8 and 16 by sex.

Figure 7 shows the osteogenic differentiation between sexes. There were no significant sex differences among the 20s, 30s, and 50s groups on Days 8 and 16 (Figure 7A,B). The quantification of the mineralization analysis on Days 8 and 16 showed no differences among the age groups (Figure 7C) and no differences between sexes (Figure 7D).

## 4. Discussion

This present study found no significant differences in the cell viability of BMSCs cultured in an osteogenic medium by age group, although a significant difference was found between sexes. Additionally, males and females showed no significant differences in the secretion of human vascular endothelial growth factor, although a significant difference was found in terms of human VEGF secretions between the 20s and 50s groups. There were no significant differences in Runx2 or collagen I expression by age or sex. Likewise, alizarin red S staining showed no significant differences in osteogenesis by age group or sex.

Many studies have shown age-related decreases in cellular proliferation or viability [19,20,21,22]. In vitro studies have reported that BMSCs display age-associated decreases in colony-forming unit-fibroblasts and bone formation [13,23]. Cells from younger individuals show more numerous proliferative precursor cells than cells from older individuals [24]. However, other in vitro studies revealed that age does not have any significant influence on the capability of cultivated human MSCs [25,26,27]. This study found no age-related differences in cell viability potential among the 20s, 30s, and 50s groups. The possible mechanism of the above findings is that BMSCs might have self-renewal capacity [26]. Another researcher suggested that body mass index is more predictive than age in males [28]. The study does not include BMSCs from very young or very old donors, but the most prominent differences may be seen in these groups.

This study found that the BMSCs from males showed a significantly lower cellular viability, compared to females, on Days 1 and 3 in osteogenic media. This was also found in a study showing that human BMSCs from female donors showed more cellular proliferation than those from male donors [26]. However, these proliferative differences either disappeared or were compensated for, and no differences in osteogenic differentiation were found between the BMSCs from male and female donors. 

This study found no differences in surface antigen SSEA-4 expression, which is regarded as a stem cell marker [29]; therefore, similar capabilities of self-renewal and mesodermal tri-lineage differentiation of the BMSCs’ niche among all age groups could be supposed. A previous report showed that the VEGF secreted by mesenchymal stem cells is involved in the differentiation of the cells, including endothelial progenitor cells [30]. This study showed that there were no significant differences between the age groups on Day 2. 

Many studies showed that BMSC subpopulations undergo osteogenic differentiation constantly, regardless of aging [19,26,27]. Osteogenic potential was present regardless of the donor’s age in vitro, although increases in donor age reduced the prevalence of bone formation, when subcutaneously implanted in nude mice [19]. Additionally, this study did not find any significant difference in osteogenesis between the 20s, 30s, and 50s age groups. However, other studies have revealed opposing results, showing that osteogenic differentiation decreased as donor age increased [13,20]. The timing used to evaluate the cell differentiation capacity and the number of samples per group may have affected the results, producing the different results, compared to other studies. The previous report showed that there were no significant differences in the relative values of adipogenesis in BMSCs for the 20s, 30s, and 50s age groups, and no obvious differences were seen between female and male groups [31]. Similarly, there were no significant differences in the chondrogenic differentiation potential of BMSCs isolated from healthy male donors vs. healthy female donors [31].

A real-time polymerase chain reaction and Western blot analysis were performed to evaluate the gene and protein expression of Runx2 and collagen I [32,33,34]. Runx2 is considered to be a critical regulator of osteoblast differentiation [35], and it regulates downstream genes that determine the osteoblast phenotype and controls the expression of osteogenic marker genes, including alkaline phosphatase [36]. Collagen I is considered as one of the bone-related proteins, and it was shown that the expression of collagen I was increased during the bone formation and was followed by mineralization-related genes, including osteocalcin [37,38].

One possible mechanism for these contradictory results is the variance between studies on the cellular passage used by the cells employed in the study [7]. The other probable mechanism is that osteogenic differentiation capacity is not influenced by donor sex or age but by several cytokines or secretory molecules [26]. The BMSCs’ function is mediated by secreted cytokines [39,40]. While the present work evaluated VEGF release, many other cytokines can affect BMSCs’ dimorphic properties [41,42]. Another study demonstrated that MSCs with a higher proliferative potential triggered a stronger osteogenic differentiation, which means that proliferative status, rather than donor age, is a dominant factor in osteogenic potential [14]. Therefore, further research would be requisite to elucidate the roles of various cytokines secreted from BMSCs and to determine whether there is an association between proliferating and differentiation potentials. Interestingly, human BMSCs have shown age-related decreases in osteogenic potential through an ALP assay, but their chondrogenic or adipogenic potential has been debatable in previous works [13,14].

This study has some limitations. First, it did not find various cytokines that could affect the differentiation potentials of human BMSCs. Second, this study did not deal with the BMSCs’ characteristics in vivo according to age or sex. Further, an in vivo study should be conducted to elucidate the roles of age or sex on human BMSCs’ differentiation potentials for therapeutic use. Various cytokines have been reported to be related to the differentiation of bone marrow-derived stem cells, including bone morphogenetic proteins and insulin-like growth factors [43,44]. Moreover, this study should be followed by a series of studies exploring the effect of cytokines on the differentiation potentials of human BMSCs.

## 5. Conclusions

In summary, the human BMSCs showed no age-related decreases in cellular viability or osteogenic differentiation potential.

## Data Availability

All data are contained within the article.
